# Diagnostic and prognostic role of long non-coding RNAs (lncRNAs) in metastatic melanoma patients with BRAF gene mutation receiving BRAF and MEK inhibitors

**DOI:** 10.1016/j.heliyon.2024.e29071

**Published:** 2024-04-01

**Authors:** Łukasz Galus, Tomasz Kolenda, Michał Michalak, Jacek Mackiewicz

**Affiliations:** a Department of Medical and Experimental Oncology, Institute of Oncology, Poznan University of Medical Sciences, Poland; b Laboratory of Cancer Genetics, Greater Poland Cancer Centre, Poznan, Poland; c Department of Computer Science and Statistics, Poznan University of Medical Sciences, Poland; d Department of Diagnostics and Cancer Immunology, Greater Poland Cancer Centre, Poznan, Poland

**Keywords:** melanoma, BRAF V600 mutation, Targeted therapy, lncRNA, biomarkers

## Abstract

Melanoma is a cancer with a high incidence rate that, despite the significant development of therapeutic options, still remains a major problem. The identification of biomarkers to select the right therapy for the right patient is one of the possibilities to improve the prognosis of patients. Potentially, the function of biomarkers could be played long non-coding RNAs (lncRNAs). The expression of selected 90 lncRNAs in serum from 30 metastatic melanoma patients with confirmed mutations in the BRAF V600 E or K gene was studied. Serum was collected prior to BRAF and MEK inhibitor therapy. The control group consisted of 16 healthy volunteers. A total of 41 lncRNAs were identified the expression of which differed statistically significantly between the patient group and the healthy volunteers. In addition, it was shown that the expression of HOXA3as (*p* = 0.033), PRINS (*p* = 0.036) and RNCR3 (*p* = 0.045) is higher in patients with the presence of CNS metastases, PFS inhibiting RNA (*p* = 0.048) is higher among patients with the presence of hepatic metastases, UCA1 (*p* = 0.008) expression is lower in patients with increased lactate dehydrogenase levels, while HOTAIRM1 (*p* = 0.044) and E2F4 antisense (*p* = 0.040) expression is lower in patients over 60 years of age. In addition, patients with high lincRNASFMBT2 expression showed longer median PFS (8.75 vs. 17.5 months, *p* = 0.0319) and OS (9.75 vs. 38 months (open observation, *p* = 0.0253). The obtained results require validation on a larger group of patients. If the results are confirmed, the indicated lncRNAs may play an important role as diagnostic and prognostic markers.

## 1 Introduction

Melanoma is a cancer with a high rate of incidence growth, which despite the significant development of therapeutic options, still remains a major problem. According to data for the United States of America, in 2021 the absolute number of new cases in the country was 106,000, and it is predicted that with the continued trend of increasing new cases and in the lack of interventions to reduce the aforementioned trend, the number of new cases in the US in 2030 will be 112,000 [1,2]. In recent years, there has been a significant breakthrough in the treatment of patients with advanced melanoma, and the medications that have been introduced are the first ever to have a proven positive effect on extending survival time. The median survival time for patients at the generalized stage before the development of targeted therapies and immunotherapies did not exceed one year, while today, during the use of the aforementioned therapies, it ranges from 13.6 to 33.6 months for BRAF inhibitor therapy in combination with a MEK inhibitor, and 72 months for ipilimumab immunotherapy with nivolumab [3,4]. Despite significant progress in the treatment of melanoma patients, more than half of those treated die. In addition to the development of prophylaxis, the emergence of new therapeutic molecules or new associations of existing therapies, the expansion of biomarker networks is also an important element. Biomarkers can help divide patients into appropriate prognostic groups and to select patients for whom a particular therapy will be effective. Such a marker could be long non-coding RNA (lncRNA). A lncRNA is a type of RNA strand, consisting of more than 200 nucleotides, that is not translated into proteins, but can modify gene function and affect various biological processes in the cell. LncRNAs are mainly found in cells, but their presence can also be found in blood serum [5,6]]. In a previous study, some of the co-authored demonstrated the importance of IGF2as, MEG3, SOX2ot and Zeb2Nat as independent prognostic biomarkers in patients receiving monotherapy with a BRAF inhibitor (vemurafenib) [6].

The aim of this study was to determine the usefulness of selected lncRNAs as diagnostic and prognostic biomarkers in melanoma patients with a BRAF V600 gene mutation and undergoing targeted therapy with BRAF inhibitors in combination with MEK inhibitors.

## 2 Materials and methods

Melanoma patients who were scheduled for BRAF and MEK inhibitor therapy (veburafenim and cobimetinib or dabrafenib and trametinib) and healthy volunteers constituting the control group were included in the study. The treatment applied was standard procedure in accordance with Polish and global guidelines. The study was observational in nature, and the only intervention was the collection of an additional blood sample. The study included patients with metastatic melanoma who were over 18 years of age and had a mutation in the BRAF V600 gene determined in an archived histopathological sample. Additionally, the patient had to be in good general condition (ECOG 0–1). Before starting treatment, the patient underwent basic laboratory blood tests and an ECG. The exclusion criterion was ECOG performance status 3–4 and abnormalities in the tests performed at grade > G2 according to CTC AE. Treatment with BRAF and MEK inhibitors was continued until disease progression/symptoms of unacceptable toxicity were observed or the patient's consent was withdrawn.The study was conducted in accordance with the ethical principles formulated in the Helsinki Declaration and relevant regulations. The Bioethics Committee of the Karol Marcinkowski University of Medical Sciences in Poznań approved the study (Resolution No. 153/16 of February 04, 2016). Everyone included in the study gave informed, written consent to participate in the project.

All subjects were taken 5 ml of blood each into tubes used for biochemical tests coated with a coagulation activator before treatment. The collected blood sample was immediately subjected to centrifugation for 10 min at 3500 rpm (Cenrifuge 5416 Eppendorf). The resulting serum was tested for hemolysis and then frozen. The tested lncRNAs are listed in Table 1.

**Table 1:** List of tested LNCRNAs.

21A	7SK	7SL (recognition particie RNA)	Air (acute insulin response)	AK023948	Alpha 280	Alpha 250	ANRIL (antisense non-coding RNA in the INK4 locus)	anti-NOS2A (antisense nitric oxide synthetase 2 enzyme)
antiPeg11 (antisense transcript to retrotransposon-like protein coding gene 11)	BACE1AS (antisense transcript of beta secretase)	BC200 (brain cytoplasmic RNA1)	CAR Intergenic 10 (chromatin associated intergenic RNA 10)	DHFR upstream transcripts (upstream transcript of disecreate human dihydrofolate reductase)	Dio3os (DIO3 opposite upstream RNA)	DISC2 (disrupted in schizophrenia 2)	DLG2AS (antisense RNA disks large homolog 2)	E2F4 antisense (antisense RNA E2F transcription factor 4)
EgoA (eosinophil granule otogeny A RNA)	EgoB (eosinophil granule otogeny B RNA)	Emx2os (empty spricles homeobox 2 opposite strand RNA)	Evf1 and Evf2	GAS5-family (lmc RNA growth arrest specific transcript 5 family)	Gomafu	H19 (H19 gene product lnc RNA)	H19 antisense	H19 upstream converved 1&2
HAR1A (highly accelerated region 1A)	HAR1B (highly accelerated region 1B)	HOTAIR (HOX antisense intergenic RNA)	HOTAIR1M (HOX antisense intergenic RNA 1 M)	HOTTIP (HOXA distal transcript antisense RNA)	Hoxa11as (homeobox A cluster 11 antistrand)	HOXA3as (homeobox A cluster 3 antistrand)	HOXA6as (homeobox A cluster 6 antistrand)	HULC (hepatocellular carcinoma up-regulated long non-coding RNA)
IGF2AS (insulin-like growth factor antisense RNA)	IPW (imprinted in Prader-Willi syndrome)	Jpx (JPX transcript)	Kcnq1ot1 (KCNQ1 opposite transcript 1)	KRASP1	L1PA16	p21	RoR	SFMBT2
VLDLR	LOC285194	LUST (Luca-15-specific transcript)	Malat1 (metastasis associated lung adenocarcinoma transcript 1)	mascRNA (MALT1 – associated small cytoplsmic RNA)	MEG3 (maternally expressed gen 3 lnc RNA)	MEG9 (maternally expressed gen 9 lnc RNA)	MER11C	ncR-uPAR
NDM29 (neuroblastoma differation marker 29)	NEAT1 (nuclear paraspeckle assembly transcript 1)	Nespas (neuroendocrine secretory protein antisense)	NRON (noncoding repressor of NAF)	NTT (noncoding transcript in T cells)	p53 mRNA	PCGEM1 (prostatę specific transcript 1)	PR antisense transcripts	PRINS
PSF inhibiting RNA	PTENP1 (phosphatase and tensin homolog pseudogene 1)	RNCR3	SAF (apoptosic mediating surface antigen FAS opposite strand)	SCA8	snaR	SNHG1 (small nuclear	SNHG3 (small nuclear RNA host gene 3)	SNHG4 (small nuclear RNA host gene 4)
SNHG5 (small nuclear RNA host gene 5)	SNHG6 (small nuclear RNA host gene 6)	sox2ot (sox overlapping transcript)	SRA (apeciffically RAC 1 associated protein)	ST7OT	TEA ncRNAs	Tmevpg1	TncRNA (tenascin-C RNA)	Tsix (TSIX transcript)
TUG1 (taurine up-regulated 1 RNA)	UCA1 (urothelial cancer associated 1 lnc RNA)	UM9-5	WT1-AS (Wilms Tumore antisense RNA 1)	Xist (X-inactive specific transcript)	Y RNA-1	Zeb2NAT (ZEB2 antisense RNA1)	Zfas1	Zfhx2as (zinc finger homebox 2, opposite strand)

Total RNA was isolated from serum samples using miRNeasy Serum/Plasma Kit (Qiagen) according to the isolation protocol for total RNA. The quality and quantity of RNA samples were checked with a NanoDrop spectrophotometer (Thermo Scientific), and samples were stored at −80 °C until use. cDNA synthesis and qRT-PCR reaction In this study, the 90 lncRNAs, potentially connected with cancer and well-annotated and registered in the lncRNA database (www.lncrnadb.org), were analyzed using the commercially available LncProfiler qPCR Array Kit (SBI). Reverse transcription was performed according to the manufacturer's protocol and was based on three steps: 1) poly-A tailing; 2) annealing anchor dT adaptor; and 3) cDNA synthesis. cDNA was used for the qRT-PCR reaction using LightCycler 480 SYBR Green I Master buffer (Roche) and lncRNA primers from Primer Plate (component of the LncProfiler qPCR Array Kit) according to the manufacturer's protocol by the LightCycler 96 (Roche). All qRT-PCR data were analyzed by calculating the ΔCt, normalized against mean expression of anti-nitric oxide synthase (NOS)2A + human accelerated region (HAR)1B + taurine upregulated gene (TUG)1, which were the most stable transcripts in all of the examined samples (healthy and cancer) with the lowest Ct s variation compared to the reference genes from the LncProfiler qPCR Array Kit (SBI). The fold-change of lncRNA expression was determined by equation 2–ΔCt and compared to the appropriate group.

Treatment was administered until disease progression (failure of therapy), unacceptable toxicity or death. If adverse effects occurred during therapy, symptomatic treatment was administered and/or treatment was interrupted and/or dose reductions were made in accordance with the drug product characteristics.

CT scans were used to evaluate treatment effect, and RECIST (Response Evaluation Criteria in Solid Tumors) version 1.1 was used to interpret the results.

First, lncRNA expression was evaluated in advanced melanoma patients compared to healthy volunteers. LncRNAs that were statistically significantly different between groups were subjected to ROC analysis in order to evaluate their diagnostic value. Subsequently, the expression of lncRNAs was evaluated and compared in subgroups formed according to selected clinical features and laboratory parameters, i.e. presence of CNS metastatic lesions, age, LDH levels, presence of hepatic metastatic lesions, number of organs involved in the neoplastic process. In the next stage, in order to determine the prognostic significance of lncRNAs, patients were divided into two groups depending on the expression level of individual lncRNAs, i.e. below the median and above the median. In such groups, PFS and OS curves were determined using the Kaplan-Meier method. Differences in PFS and OS values between the analyzed groups were assessed by Log-rank test. Further univariate and multivariate Cox regression analysis was conducted to determine the factors influencing the risk of progression or death. LncRNAs that showed statistical significance in previous analyses were evaluated for association with selected clinical and laboratory factors, i.e., age, gender, LDH levels CNS metastasis, hepatic metastasis, and number of organs involved in the tumor process. Normality of the data distribution was established using the Shapiro-Wilk test, and the Mann-Whitney test was used for the comparatives analysies. Statistical analysis was performed with STATA 17 - StataCorp. 2021. Stata Statistical Software: Release 17. College Station, TX: StataCorp LLC. All tests were considered significant at p < 0.05.

## 3 Results

### 3.1 Patients

The study included 30 patients undergoing combination targeted therapy with a BRAF inhibitor combined with a MEK inhibitor. Three patients had a MEK inhibitor added to the BRAF inhibitor due to treatment reimbursement limitations, with a delay of 1.5, 7 and 10 months, respectively. By the time of the current outcome analysis, treatment had been terminated in 28 patients, including 27 due to disease progression or death, and 1 patient due to unacceptable toxicity in the form of recurrent hyperbilirubinemia. After disease progression, 16 patients started second-line treatment with anti-PD-1 antibodies, i.e. nivolumab or pembrolizumab. Out of the total, 8 patients are alive, of which treatment is continued in 2, and the remaining 6 receive second-line treatment, i.e. anti-PD-1 immunotherapy. Detailed characteristics of the patients and treatment results are provided in Table 2.

**Table 2:** Characteristics of the patients included in the study.

number of patients	30				
LDH concetrations	LDH ≤ ULN			LDH > ULN	
	12 (40%)			18 (60%)	
feature „M″	M1a		M1b	M1c	M1d
	4 (13.3%)		4 (13.3%)	9 (30%)	13 (43.3%)
numer of organs with metastases	range			median	
	1–8			3	
age	median (years)			range (years)	
	60.5			25–82	
sex	men			women	
	14 (46.7%)			16 (53.3%)	
treatment	vemurafenib, cobimetynib			dabrafenib, trametynib	
	18 (60%)			12 (40%)	
treatment response	ORR			PD	
	28 (93.3%)			2 (6.7%)	
	CR	PR			
	10% (3)	83.3% (25)			
median PFS (range)	10.25 months (2–47.5 [open observation])				
median OS (range)	19.1 months (3.25–49.25 [open observation])				

The control group included 16 adult volunteers (10 women and 6 men) aged 27–57 years, without a history of chronic diseases.

### 3.2 Differences in serum lncRNA expression between melanoma patients with a BRAF gene mutation and healthy volunteers

The study showed a statistically significant difference in the expression of 41 lncRNAs between a group of patients and a group of 16 healthy volunteers. Overexpression was found in case of 35 lncRNAs, and reduced expression was observed in case of 6 lncRNAs. Detailed results are shown in Table 3.

**Table 3:** Expression lncRNA (median expression [IQR – interquartile range)) showing a statistically significant difference between patients with melanoma and healthy volunteers.

lncRNA	median expression in healthy volunteers	median expression in patients with melanoma before iBRAF/MEK therapy	*p* value
7SK	0.00085 [0.0002731–0.01552]	7.88 [0.299–51.93]	<0.001
Air	0.0006261 [0.0001743–0.004186]	0.4807 [0.001305–2.611]	0.002
AK023948	0.00188 [0.0002731–0.5422]	6.729 [0.9575–78.7]	<0.001
Alpha 250	14.22 [4.499–269.4]	0.0832 [0.0004677–7.681]	<0.001
anti-NOS2A	49.48 [11.25–97.82]	0.297 [0.03571–101]	0.011
DISC2 (family)	0.238 [0.0007006–2.114]	2.222 [0.3888–8.45]	0.011
EGO B	28 [2.348–145.9]	88.65 [33.64–881.1]	0.039
Emx2os	0.5564 [0.01719–1.74]	5.78 [0.7438–29.09]	0.001
Gomafu	0.4986 [0.02786–1.92]	7.093 [1.154–14.41]	0.001
H19	3.732 [0.006757–30.42]	20.41 [2.298–91.02]	0.021
HOTAIR	1003 [178.8–6851]	0.5253 [0.1409–106.1]	<0.001
HOTAIRM1	1.536 [0.109–11.9]	32.46 [9.816–447.7]	0.001
HOXA3as	0.003923 [0.0005053–0.379]	0.2879 [0.006312–0.9031]	0.010
HULC	0.3897 [0.002013–2.119]	2.978 [0.4758–9.076]	0.006
Jpx	0.03263 [0.0005053–0.511]	1.901 [0.1676–9.547]	0.003
Kcnq1ot1	1.452 [0.0005451–6.703]	8.795 [1.064–16.94]	0.049
KRASP1	0.4624 [0.007737–3.597]	4.276 [0.8849–10.96]	0.002
lincRNA-p21	2.212 [1.133–38.26]	32.11 [2.496–76.35]	0.044
lincRNA-VLDLR	22696 [9766–85238]	8.054 [2.177–6137]	<0,001
LUST	0.005258 [0.0003305–38.69]	694.3 [148.9–5758]	0.001
mascRNA	0.6371 [0.01463–1.899]	13.05 [0.6325–110.5]	0.002
MEG3 (family)	0.003923 [0.0005053–0.06665]	0.5845 [0.0461–3.554]	0.001
MER11C	0.4566 [0.001224–1.488]	10.25 [1.699–52.06]	0.000
ncR-uPAR	9.061 [0.03438–112.1]	235.1 [8.317–818.9]	0.002
p53 mRNA	0.0005548 [0.0001614–0.003985]	2.723 [0.7846–7.605]	0.000
PCGEM1	0.007462 [0.000527–2.56]	2.345 [0.2417–7.124]	0.011
PR antisense transcripts	0.0006696 [0.0001833–0.09748]	0.1492 [0.03316–0.8071]	0.002
PRINS	0.00085 [0.0002545–0.005561]	0.1835 [0.005722–1.528]	0.000
PSF inhibiting RNA	0.0005113 [0.0001614–0.00173]	0.01514 [0.001649–0.3236]	0.001
PTENP1	0.0006696 [0.0001791–0.005095]	0.0824 [0.003714–0.4357]	0.001
RNCR3	1.511 [0.08878–7.486]	11.39 [2.362–38.98]	0.002
SAF	0.4671 [0.03929–6.046]	2.87 [0.746–22.36]	0.023
SNHG4	55.34 [9.952–132.2]	1467 [117.6–6600]	0.000
SNHG5	0.08438 [0.001042–0.7702]	0.5834 [0.03067–1.589]	0.044
Sox2ot	0.0114 [0.0005451–2.465]	1.033 [0.06938–2.535]	0.031
TEA ncRNAs (family)	0.3677 [0.00064–1.619]	1.523 [0.4984–5.394]	0.029
TUG1 (family)	31.57 [18.55–176.3]	15.26 [2.792–82.23]	0.033
UCA1	0.001477 [0.0002605–0.2411]	0.09125 [0.006312–0.7552]	0.035
Xist	0.03913 [0.00064–0.7843]	3.75 [0.2192–12.46]	0.001
Zeb2NAT	0.3109 [0.0007895–5.261]	7.353 [1.15–21.75]	0.001
Zfhx2as	2512 [379.7–30847]	2.126 [0.2767–37.61]	<0.001

All 41 lncRNAs in the ROC analyses conducted showed diagnostic properties, and for the estimated cutoff values, sensitivity levels ranged from 53% to 100%, while specificity levels ranged from 44% to 100%. The above results are shown in Table 4.

**Table 4:** Sensitivity and specificity of lncRNA as biomarkers in melanoma patients and healthy volunteers based on the ROC analysis.

lncRNA	Cut off expression lnc	AUC [95%CI]	sensitivity (%)	specificity (%)
7SK	0.03435	0.84 [0.70–0.97]	88	87
Air	0.0059	0.78 [0.64–0.91]	70	88
AK023948	0.0059	0.88 [0.77–0.98]	97	69
Alpha 250	0.1668	0.82 [0.70–0.94]	94	57
anti-NOS2A	1.3050	0.73 [0.58–0.88]	100	63
DISC2 (family)	0.3340	0.73 [0.57–0.89]	77	63
EGO B	38.6393	0.69 [0.51–0.86]	70	69
Emx2os	1.9399	0.81 [0.68–0.93]	67	81
Gomafu	2.0936	0.81 [0.68–0.93]	67	81
H19	0.0763	0.71 [0.55–0.87]	97	44
HOTAIR	40.1131	0.88 [0.78–0.97]	94	77
HOTAIRM1	6.5796	0.83 [0.71–0.96]	80	75
HOXA3as	0.0138	0.73 [0.57–0.90]	73	75
HULC	0.6897	0.75 [0.59–0.91]	73	75
Jpx	0.5736	0.77 [0.61–0.92]	70	81
Kcnq1ot1	6.7928	0.68 [0.51–0.86]	53	81
KRASP1	0.7101	0.78 [0.63–0.92]	80	69
lincRNA-p21	7.3411	0.69 [0.53–0.84]	63	75
lincRNA-VLDLR	3031.8864	0.87 [0.76–0.97]	94	73
LUST	53.5198	0.81 [0.68–0.94]	80	88
mascRNA	11.3451	0.78 [0.65–0.91]	53	100
MEG3 (family)	0.0256	0.80 [0.65–0.95]	80	75
MER11C	2.0792	0.87 [0.76–0.98]	70	94
ncR-uPAR	192.4047	0.78 [0,64-0,91]	53	94
p53 mRNA	0.4918	0.93 [0.85–1.00]	83	100
PCGEM1	0.0857	0.73 [0.55–0.91]	93	63
PR antisense transcripts	0.0028	0.77 [0.62–0.93]	87	69
PRINS	0.0159	0.85 [0.73–0.96]	70	94
PSF inhibiting RNA	0.0010	0.80 [0.67–0.94]	80	75
PTENP1	0.0028	0.79 [0.64–0.94]	80	75
RNCR3	13.5104	0.78 [0.64–0.92]	50	94
SAF	0.5007	0.71 [0.53–0.89]	83	56
SNHG4	79.7825	0.83 [0.71–0.95]	83	75
SNHG5	0.0059	0.68 [0.50–0.86]	93	50
Sox2ot	0.0334	0.70 [0.51–0.88]	83	63
TEA ncRNAs (family)	0.1424	0.70 [0,52-0,88]	93	50
TUG1 (family)	14.3403	0.69 [0.54–0.84]	94	50
UCA1	0.0020	0.69 [0.51–0.88]	87	56
Xist	0.8938	0.81 [0.67–0.94]	73	81
Zeb2NAT	0.3191	0.79 [0.64–0.94]	97	56
Zfhx2as	114.0877	0.84 [0.70–0.98]	88	87

LncRNA – non-coding RNA; ROC – Reciver Operating Statistic. AUC – area under the curve; CI – confidence interval).

### 3.3 Differences in lncRNA expression in the serum of melanoma patients with a BRAF gene mutation according to the presence of known clinical prognostic factors

Further analysis showed a difference in the expression level of lncRNAs in the serum of melanoma patients depending on the presence of CNS metastasis and hepatic metastasis. Serum in the above patients was collected before treatment. The expression of HOXA3as (*p* = 0.033), PRINS (*p* = 0.036) and RNCR3 (*p* = 0.045) was higher in patients with the presence of CNS metastases, and the expression of PFS inhibiting RNA (*p* = 0.048) was higher in patients with hepatic metastases. Subsequent results indicated a difference in lncRNA expression in groups formed according to LDH concentration and age. Thus, the expression of UCA1 (*p* = 0.008) is lower in patients with increased LDH levels, while HOTAIRM1 (*p* = 0.044) and E2F4 antisense (*p* = 0.040) are lower in patients older than 60 years. However, there were no statistically significant differences in lncRNA expression between patients with tumor process involving <3 organs and patients with ≥3 organs involved. Detailed results are included in Table 5.

**Table 5:** Comparison of lncRNA expression level [median expression, [interquartile range (IQR)] depending on clinical prognostic factors in patients with advanced melanoma.

lncRNA expression depending on the presence of CNS metastases			
lncRNA	LncRNA expression in patients without CNS metastases	LncRNA expression in patients with CNS metastases	p value
HOXA3as	0.04222 [0.001409–0.6262]	0.725 [0.1418–2.556]	0.033
PRINS	0.06233 [0.001157–0.8449]	0.6426 [0.1066–2.761]	0.036
RNCR3	4.5 [1.269–24.13]	25.39 [5.671–65.72]	0.045
**lncRNA expression depending on the presence of liver metastases**			
lncRNA	LncRNA expression in patients without liver metastases	LncRNA expression in patients with liver metastases	p value
PSFinhibitingRNA	0.00341 [0.007269–0.6105]	0.1482 [0.0002469–0.06241]	0.048
**lncRNA expression depending on LDH**			
lncRNA	LncRNA expression in patients with LDH normal	LncRNA expression in patients with LDH above normal	p value
UCA1	0.367 [0.05246–0.9054]	0.02219 [0.002431–0.1857]	0,008
**LncRNA expression depending on age**			
lncRNA	LncRNA expression in patients up to 60 years old (inclusive)	LncRNA expression in pacjent more than 60 years old	p value
HOTAIRM1	78.95 [28.64–36410]	24.63 [4.975–40.71]	0.044
E2F4 antisense	21.55 [0.2716–369.9]	0.6248 [0.2011–2.94]	0.040

### 3.4 Significance of lncRNA expression in the serum of melanoma patients with a BRAF gene mutation undergoing BRAFi and MEKi therapy as a biomarker of prognostic significance

**Fig. 1:**
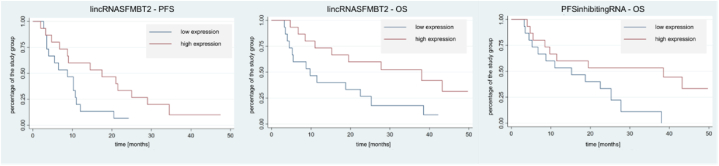
Kaplan-Meier curves of lncRNA showing significant statistical differences in PFS/OS in groups composed according to their expression.

In order to determine the prognostic value of the tested lncRNAs, patients were divided into a group presenting low (below median) and high (above median) expression of 90 evaluated lncRNAs. The groups thus formed were compared for differences in PFS and OS. Patients with low expression of lincRNASFMBT2 had a median PFS of 8.75 months [95% CI (3.5–10.75)], while those in the high expression group had a median PFS of 17.5 months [95% CI (4.75–25)]; *p* = 0.0319. Median OS was 9.75 months [95% CI (4–22.5)] and 38 months (open observation), respectively; *p* = 0.0253. For PFS inhibiting RNA, there was no difference in PFS between the study groups (*p* = 0.051), while median OS in the group of patients presenting lower expression was 15.25 months [95% CI (4.5–25.25)], which was lower than in the group with high expression, where it was 38 months (open observation); *p* = 0.0361. Kaplan-Meier curves showing PFS and OS for the above results are shown in Fig. 1. For the other lncRNAs, there was no statistically significant difference in PFS and OS, between the groups with low and high expression of lncRNAs studied. The statistical significance of obtained results for lincRNASFMBT2 and PFS inhibiting RNAs was confirmed by univariate Cox regression analysis, while it was not confirmed by multivariate analysis. The results are presented in Table 6. The Mann-Whitney test conducted to confirm the independent prognostic significance of lncRNAs in question showed that factors, which may influence the course of the disease, i.e. age, gender, LDH levels, CNS metastases or the number of organs involved in the tumor process, are not associated with high expression of both lincRNASFMBT2 and PFS inhibiting RNAs, while the presence of hepatic metastases in accordance to one of the previous analyses is associated with higher expression of PFS inhibiting RNAs. Detailed results are presented in Table 7, Table 8.

**Table 6:** Univariate and multivariate analysis.

		OS						PFS					
Parametr	subgrups	univariate analysis			multivariate analysis			univariate analysis			multivariate analysis		
		HR	95%CI	*p*	HR	95%CI	*p*	HR	95%CI	*p*	HR	95%CI	*p*
age	≤60 vs > 60	1.25	0.54–2.91	0.607	–	–	–	1.11	0.51–2.40	0.26	–	–	–
stage	III,M1a,M1b vs M1c, M1d	4.76	1.39–16.30	**0.013**	–	–	–	3.02	1.18–7.72	**0.021**	–	–	–
CNS metastases	no vs present	5.01	1.92–13.09	**0.001**	5.4	1.80–16.25	**0.003**	2.89	1.197,02	**0.019**	12.51	2.13–73.49	**0.005**
liver metastases	no vs present	2.66	1.10–6.45	**0.03**	–	–	–	3.2	1.40–7.36	**0.006**	–	–	–
sex	W vs M	2.5	0.93–6.71	0.069	–	–	–	1.39	0.64–3.03	0.411	–	–	–
LDH	≤ULN vs > ULN	1.05	0.45–2.47	0.909	–	–	–	1.11	0.51–2.43	0.26	–	–	–
numbers of organs with metastases	<3 vs ≥ 3	5.86	2.18–15.75	**<0.001**	6.15	2.13–17.78	**0.001**	6.37	2.42–16.77	**<0001**	68.28	9.90–471.14	**<0.001**
lincRNASFMBT2	low expression vs high expression	0.38	0.15-0,92	**0.031**	–	–	–	0.41	0.18–0.95	**0.038**	–	–	–
PSFinhibitingRNA	low expression vs high expression	0.38	0.15–0.97	**0.043**	–	–	–	–	–	–	–	–	–

**Table 7:** Relationship of PFSinhibitingRNA concetration (median and quartole range) with clinical feature and laboratory parametrs.

Clinical feature/laboratory parametr		number of patients	PFSinhibitingRNA			*p* value
			median	lower quartil	upper quartil	
age	≤60	16	0.03626	0.00152	0.2779	0.917
	>60	14	0.01372	0.00292	0.5459	
sex	men	16	0.00630	0.00055	0.2277	0.253
	women	14	0.09709	0.00371	0.7519	
LDH concetration	≤norm	13	0.1530	0.00375	0.4732	0.503
	>norm	17	0.00532	0.00158	0.2256	
CNS metastases	no	17	0.02017	0.00024	0.2848	0.380
	yes	13	0.01011	0.00457	0.4732	
liver metastases	no	19	0.1482	0.0072	279.3	**0.048**
	yes	11	0.00341	0.00024	0.0624	
numer of organs with metastases	<3	15	0.153	0.00727	0.6105	0.082
	≥3	15	0.00414	0.00145	0.0624

**Table 8:** Relationship of lincRNASFMBT2 concetration (median and quartole range) with clinical feature and laboratory parametrs.

Clinical feature/laboratory parametr		Numer of patients	lincRNASFMBT2			*p* value
			median	lower quartil	upper quartil	
age	≤60	16	0.03448	0.00095	147.5	0.394
	>60	14	0.3359	0.00799	59.42	
sex	men	16	0.1715	0.00543	6.801	0.253
	women	14	0.08718	0.00012	335.7	
LDH concetration	≤norm	13	0.00868	0.00008	0.8595	0.072
	>norm	17	0.6355	0.00756	252.7	
CNS metastases	no	17	0.2724	0.00756	247.3	0.195
	yes	13	0.00868	0.00027	1.718	
liver metastases	no	19	0.6355	0.00463	204.8	0.212
	yes	11	0.0105	0.0005	0.3994	
numer od organs with metastases	<3	15	0.6355	0.00463	194	0.384
	≥3	15	0.0185	0.0005	2.801	

## 4 Discussion

Discovery and subsequent validation of appropriate biomarkers would allow, among others, early diagnosis of melanoma, precise evaluation of stage, detection of recurrence after radical surgical treatment, as well as optimal selection of therapy for advanced disease and monitoring of the effectiveness of ongoing treatment. The function of such a biomarker could potentially be performed by ncRNAs (non-coding RNAs). Their presence in blood serum offers the possibility of simple, minimally invasive and reproducible obtaining material for analysis.

As shown in our results, the expression profile of some lncRNAs differs between advanced melanoma patients and healthy individuals. In the future, once the results obtained are validated, the creation of such a panel may facilitate the diagnosis of disseminated disease.

One of the lncRNAs that showed significantly higher expression in the serum of melanoma patients is H19. Previous reports have described higher expression of lncRNA in question in melanoma cells and at the same time found a correlation between its higher expression and worse prognosis [7].

Significantly higher expression in the serum of patients was also found for Kcnq1ot1. Analogous results have already been described for expression in melanoma tissues and cells. According to literature reports, KCNQ1OT1 promotes cell proliferation and its ability to metastasize [8].

The study also found significantly higher serum SNHG5 expression in melanoma patients compared to healthy volunteers. Previously, higher SNHG5 lncRNA expression has been described in melanoma cell lines and tissues. Higher SNHG5 expression has also been shown to be associated with higher melanoma stage. Blocking the function of SNGH5 promoted apoptosis and inhibited cell proliferation, as well as reduced the ability of cells to become invasive [9].

Another lncRNA the expression of which was statistically significantly higher in patients’ serum is XIST. To date, in vitro studies conducted on A375 melanoma cells have revealed overexpression of XIST and a positive effect of lncRNA in question on cell proliferation and viability, as well as promotion of oncogenesis [10,11].

Uca1 lncRNA was also distinguished by its overexpression in the serum of patients compared to healthy subjects. Previously available literature described its increased expression in melanoma cells, as well as their reduced ability to migrate after Uca1 silencing. Interestingly, Uca1 showed higher expression in tissue from melanoma patients in stage 3 and 4 compared to stage 1 and 2 [12].

Another three lncRNAs, i.e. HOTIAR, MEG3 and TUG1 (family), are of particular interest, as the results obtained in the current study appear to be different from previous literature reports.

In case of the former, among others, its moderate expression in the tissues of benign nevi and primary foci of melanoma that have not spread was described, compared to its very high expression in primary foci and metastases derived from them. In vitro, HOTAIR silencing has been shown to be associated with reduced potential for melanoma cell migration and invasiveness [12,13]. HOTAIR expression was also found in lymphocytes inside the tumor, and the expression decreased in lymphocytes further from the tumor. Its presence in lymphocytes within the tumor may suggest a role in modulating the tumor microenvironment [14]. HOTAIR promotes the growth and metastasis of melanoma cells. Overexpression of the lncRNA in question has also been described in cases of other cancers [15]. In the current analysis, the lncRNA in question showed, differently from all the above examples, a statistically significant lower expression among melanoma patients, compared to healthy volunteers.

A reduced expression in the serum of patients was also shown for the TUG1 (family) lncRNA in the current analysis, while results published so far show its increased expression in melanoma cell lines. The available literature showed that inhibition of TUG1 (family) function resulted in inhibition of melanoma cell growth, as well as their ability to metastasize (tested using heterotransplantation in animal models) [16].

MEG3, according to literature reports, is responsible for the inhibition of tumor growth and metastasis by modulating the miR-21/E-cadherin axis [17]. In contrast, in our analysis, MEG3 expression was statistically significantly higher in the serum of patients than in the serum of healthy subjects.

The reasons for different than expected results for HOTIAR, MEG3 and TUG1 (family) are unknown, but the different material analyzed (tumor tissue vs serum) should be taken into account. It should also be noted that the source of lncRNAs can be not only the tumor, but also cells of the immune system. Therefore, the expression of a given lncRNA may be related not only to the tumor biology, but also to the efficiency of immune system. Different patient populations may also be an important reason. Some of the literature reports are based on studies performed in the Chinese population, while the study discussed here was conducted among Caucasian patients. In order to clarify the significance of HOTIAR, MEG3 and TUG1 (family), it would be advisable to conduct an analysis on a large population of melanoma patients and healthy patients, both in tumor tissue and serum.

Finally, literature data are also available regarding the role of selected lncRNAs the different expression of which plays a role in melanoma development, and in our study, these differences were found to be statistically insignificant. Thus, the expression of GAS5 in the results of present study did not differ statistically significantly between melanoma patients and healthy volunteers. However, in the available literature, its overexpression is related to decreased expression of MMP2, a protein associated with ECM (extracellular matrix) degradation, which consequently reduces the ability of melanoma cells to migrate and invade [10,18]. Other lncRNAs influencing epithelial-mesenchymal transition (EMT) include BRAF-activated lncRNA (BANCR) which is overexpressed in melanoma and thus plays a potential functional role in melanoma cell migration. Based on studies performed on colorectal cancer cells, it was demonstrated that BANCR induces (EMT) through a kinase-dependent mechanism regulated by MEK/extracellular signaling. During treatment with MEK inhibitor - U0126 reduced migration in cells overexpressing BANCR. However, BANCR expression was not assessed in this study [19,20].Other lncRNAs that have been described to play a role in promoting melanoma development, and in the current analysis did not show a statistically significant difference, include MALT1 (overexpressed in melanoma tissue) and Anril [12].

CNS metastasis is an important biomarker with negative prognostic significance. Based on our analysis, patients with CNS metastases had statistically significantly higher expression of 3 lncRNAs i.e.: HOXA3as, PRINS and RNCR3. Their role in melanoma patients has not been previously described. Also, reports on their function in other cancers are limited.

Another organ the invasion of which is a negative prognostic factor is the liver. Out of 90 lncRNAs, only 1 i.e. PSF inhibiting RNA showed statistically significantly lower expression in patients with the presence of metastases in the organ in question. So far, other lncRNAs associated with such localization of metastatic lesions are unknown. There are also no data on the significance and mechanism of PFS inhibiting lncRNAs action in melanoma or other cancers. Repeating the study on a larger group will perhaps allow the future use of PSF inhibiting RNA as a biomarker in diagnosing hepatic metastases. Such an assayable serum marker could be helpful, for example, in the case of lesions that are difficult to evaluate unequivocally on imaging studies, e.g., non-measurable lesions, i.e., <1 cm, in the lack of previous results to determine growth dynamics.

LDH levels are also of important prognostic significance in determining the disease stage. Out of the 90 lncRNAs analyzed, only UCA1 showed statistically significant higher expression in patients with normal LDH. In contrast, previous literature has described increased expression of lncRNA in question in melanoma cells, among others [12].

Approximately 90% of melanoma patients experience a complete or partial response or disease stabilization as a result of treatment with BRAF and MEK inhibitors [21,22]. About 10% of patients, on the other hand, are found to have primary progression. Due to the large disproportion in the number of patients forming both mentioned subgroups in our study, i.e. 28 (93%) vs. 2 (7%), the analyses comparing lncRNA expression according to the type of response were not conducted. In the previous paper of our co-authorship, we presented results showing a correlation of primary resistance to treatment with overexpression of 7SL and Zeb2Nat as well as reduced expression of Zfas1 and AIR. Also inversely, overexpression of Zfas1 and AIR and lower expression of 7SL and Zeb2NAT were related to achieving an objective response to the applied targeted therapy [6]. To date, the only paper evaluating the role of lncRNAs as prognostic biomarkers during targeted therapy for patients with advanced melanoma is the aforementioned 2019 paper by our co-author, which investigated the expression of selected lncRNAs in the plasma of patients with metastatic melanoma. This study showed the importance of IGF2as, MEG3, SOX2ot and Zeb2Nat as independent prognostic biomarkers in patients receiving monotherapy with the BRAF inhibitor (vemurafenib). Higher expression of IGF2as, MEG3 and SOX2ot and lower expression of Zeb2Nat were related to statistically significantly longer median OS. In contrast, higher MEG3 expression and lower Zeb2Nat expression were additionally associated with longer median PFS. So far, the function of the above-mentioned circulating plasma lncRNAs has not been described [6]. It should be noted that the study presented above also evaluated the prognostic significance of lincRNASFMBT2 lncRNA and PFS inhibiting lncRNA. No statistical significance was found for either of the two. The differences in obtained results in the above-mentioned publication with the present paper may be due to the different therapies used in both studies, i.e. iBRAF monotherapy and combination therapy of iBRAF and iMEK. In addition, in the study published in 2019, lncRNA expression was assessed in the plasma of patients, while in the present study it was assessed in serum. Equally important, in the previous study, some patients received targeted therapy as a part of their next line of treatment, characterized by a higher percentage of patients with CNS metastases and a lower percentage with increased LDH.

## 5 Conclusions

The above analysis is the first report on the usefulness of circulating lncRNAs in serum, as potential prognostic biomarkers in advanced melanoma patients undergoing iBRAFi and iMEK combination therapy, and at the same time the second study of lncRNAs, in advanced melanoma patients undergoing targeted therapy. The lncRNA panel presented, after validation, may allow to differentiate a group of advanced melanoma patients from healthy volunteers. In addition, HOXA3as, PRINS, RNCR3 after validation may provide a diagnostic biomarker for the diagnosis of central nervous system metastasis, PSF inhibiting RNA may provide a diagnostic biomarker for the diagnosis of hepatic metastasis, and the expression profile of selected lncRNAs may allow differentiation of patient groups according to age (HOTIARM1 and E2F4) and LDH levels (UCA1). The significance of lincRNASFMT2 expression as a prognostic biomarker is uncertain. In univariate analysis lincRNASFMT2 showed statistical significance, but multivariate analysis did not confirm the previously obtained result. The prognostic significance of lincRNASFMT2 requires study in a larger patient population.

In addition to the aforementioned validation, further randomized studies would be recommended in order to determine the predictive value of the tested lncRNAs. Such an analysis would allow to select patients among whom iBRAF and iMEK therapy will be effective.

## Ethics approval and consent to participate

The Bioethics Committee of the Karol Marcinkowski University of Medical Sciences in Poznań approved the study (Resolution No. 153/16 of February 04, 2016). Everyone included in the study gave informed, written consent to participate in the project.

## Funding

This work was supported by the 10.13039/501100004442National Science Centre, Poland, allocated on the basis of decision no.: 2016/21/B/NZ7/01773.

## Data availability statement

Data will be made available on request.

## CRediT authorship contribution statement

**Łukasz Galus:** Writing – original draft, Visualization, Project administration, Methodology, Data curation, Conceptualization. **Tomasz Kolenda:** Methodology, Investigation, Conceptualization. **Michał Michalak:** Software, Formal analysis, Data curation. **Jacek Mackiewicz:** Writing – review & editing, Supervision, Methodology, Funding acquisition, Conceptualization.

## Declaration of competing interest

The authors declare the following financial interests/personal relationships which may be considered as potential competing interests: Lukasz Galus reports a relationship with 10.13039/100002491Bristol-Myers Squibb Co that includes: speaking and lecture fees and travel reimbursement. Jacek Mackiewicz reports a relationship with 10.13039/100002491Bristol-Myers Squibb Co that includes: speaking and lecture fees and travel reimbursement. Jacek Mackiewicz reports a relationship with 10.13039/100004334Merck & Co Inc that includes: speaking and lecture fees and travel reimbursement. Lukasz Galus reports a relationship with 10.13039/100013226Pierre Fabre SA that includes: speaking and lecture fees and travel reimbursement. Jacek Mackiewicz reports a relationship with 10.13039/100013226Pierre Fabre SA that includes: speaking and lecture fees and travel reimbursement. Lukasz Galus reports a relationship with 10.13039/100008272Novartis Pharmaceuticals Corporation that includes: speaking and lecture fees and travel reimbursement. Jacek Mackiewicz reports a relationship with 10.13039/100008272Novartis Pharmaceuticals Corporation that includes: speaking and lecture fees and travel reimbursement. Lukasz Galus reports a relationship with 10.13039/100004334Merck & Co Inc that includes: speaking and lecture fees and travel reimbursement. Jacek Mackiewicz reports a relationship with 10.13039/100004337Roche that includes: speaking and lecture fees and travel reimbursement. If there are other authors, they declare that they have no known competing financial interests or personal relationships that could have appeared to influence the work reported in this paper.
